# PReS-FINAL-2012: Introducing a new approach to clinical care of juvenile dermatomyositis: the juvenile dermatomyositis multidimensional assessment report

**DOI:** 10.1186/1546-0096-11-S2-P25

**Published:** 2013-12-05

**Authors:** GC Varnier, C Ferrari, A Consolaro, D Marafon, C Pilkington, S Maillard, M Jelusic Drazic, S Dalpra', A Civino, A Martini, A Ravelli

**Affiliations:** 1 pediatria II, IRCCS G. GASLINI, Genova, Italy; 2 Rheumatology, Great Ormond Street hospital for Children, London, UK; 3 Pediatrics, University Hospital, Zagreb, Croatia; 4 Pediatria, Azienda Ospedaliera Card, Panico, Tricase (LE), Italy

## Introduction

In recent years, there has been an increasing interest in parent/child-reported outcomes (pcros) in pediatric rheumatology practice. Incorporation of these measures in patient assessment is deemed important as they reflect the parents' and children's perception of the disease course and the effectiveness of therapeutic interventions. Although several measures of single pcros have been developed, to date a clinical measure that groups all pcros used in the assessment of children with juvenile dermatomyositis (JDM) does not exist. Such measure would provide a physician with a thorough and systematic overview of the patient status to be scanned at the start of the visit. This would facilitate focus on matters that require attention, leading to more efficient and effective clinical care.

## Objectives

To develop and test a new multidimensional questionnaire incorporating all main pcros to be used in the assessment of children with JDM in standard clinical care.

## Methods

The Juvenile Dermatomyositis Multidimensional Assessment Report (JDMAR) includes 15 parent/child-centered measures that assess well-being, pain, functional status, health-related quality of life, fatigue, disease activity, disease status and course, disease manifestations, side effects of medications, therapeutic compliance, problems at school, and satisfaction with illness outcome. The JDMAR is proposed for use as both proxy-report and patient self-report, with the suggested age range of 7-18 years for use as self-report.

## Results

A total of 107 consecutive JDM patients (44 male, 63 female) seen in 4 pediatric rheumatology centers (2 in Italy, 1 in UK and 1 in Croatia) were included in the study. Median disease duration was 3,1 years (IQR:1,0-5,8) and median age at visit was 10 years (IQR:6,4-13,6). All parents and children reported that the questionnaire was simple and easy to understand. Completion and scoring appeared to be quick, requiring 5-10 minutes. The proportion of parents who reported normal scores on the various JDMAR scales are summarized in the table.

**Table 1:** 

JDMAR assessments On a 10-cm VAS	Pts assessed	No. Positive (%)	Others JDMAR assessments	Pts assessed	No. Positive (%)
Normal well-being	98	39 (39.8)	Normal functional ability	97	53 (54.6)

No pain	99	52 (53.6)	Normal HRQL - Total score	94	27 (28.7)

No fatigue	73	24 (32.9)	Remission	92	50 (54.3)

No disease activity	67	31 (46.3)	Satisfied with illness outcome	93	66 (71.0)

## Conclusion

Development of the JDMAR provides a promising approach to quantitative measurement in standard pediatric rheumatology care. Availability of this new instrument may foster regular use of parent/patient questionnaires in routine practice and contribute to improved quality of care of children with JDMAR.

## Disclosure of interest

None declared.

